# The Dopamine Prediction Error: Contributions to Associative Models of Reward Learning

**DOI:** 10.3389/fpsyg.2017.00244

**Published:** 2017-02-22

**Authors:** Helen M. Nasser, Donna J. Calu, Geoffrey Schoenbaum, Melissa J. Sharpe

**Affiliations:** ^1^Department of Anatomy and Neurobiology, University of Maryland School of Medicine, BaltimoreMD, USA; ^2^Cellular Neurobiology Research Branch, National Institute on Drug Abuse Intramural Research Program, BaltimoreMD, USA; ^3^Solomon H. Snyder Department of Neuroscience, Johns Hopkins University, BaltimoreMD, USA; ^4^Princeton Neuroscience Institute, Princeton University, PrincetonNJ, USA

**Keywords:** prediction error, attention, associative learning, dopamine, model-based learning

## Abstract

Phasic activity of midbrain dopamine neurons is currently thought to encapsulate the prediction-error signal described in Sutton and Barto’s (1981) model-free reinforcement learning algorithm. This phasic signal is thought to contain information about the quantitative value of reward, which transfers to the reward-predictive cue after learning. This is argued to endow the reward-predictive cue with the value inherent in the reward, motivating behavior toward cues signaling the presence of reward. Yet theoretical and empirical research has implicated prediction-error signaling in learning that extends far beyond a transfer of quantitative value to a reward-predictive cue. Here, we review the research which demonstrates the complexity of how dopaminergic prediction errors facilitate learning. After briefly discussing the literature demonstrating that phasic dopaminergic signals can act in the manner described by Sutton and Barto (1981), we consider how these signals may also influence attentional processing across multiple attentional systems in distinct brain circuits. Then, we discuss how prediction errors encode and promote the development of context-specific associations between cues and rewards. Finally, we consider recent evidence that shows dopaminergic activity contains information about causal relationships between cues and rewards that reflect information garnered from rich associative models of the world that can be adapted in the absence of direct experience. In discussing this research we hope to support the expansion of how dopaminergic prediction errors are thought to contribute to the learning process beyond the traditional concept of transferring quantitative value.

## Introduction

The discovery that midbrain dopaminergic neurons exhibit a strong phasic response to an unexpected reward which subsequently transfers back to a cue which predicts its occurrence has been revolutionary for behavioral neuroscience (Schultz, 1997; Schultz et al., 1997). This was in part because this pattern of firing mimics the teaching signal predicted to underlie learning in models of reinforcement learning (Bush and Mosteller, 1951; Rescorla and Wagner, 1972; Mackintosh, 1975; Pearce and Hall, 1980; Sutton and Barto, 1981). The key concept in these learning models is that learning about reward-predictive cues is regulated by prediction error. When a subject experiences a reward that they did not anticipate in the presence of a cue, a prediction error is elicited to drive learning so that the antecedent cue comes to motivate behavior directed toward the outcome. This prediction error is generally conceptualized as a quantitative discrepancy between the outcome expected when the cue was presented, and the outcome that was actually experienced (Bush and Mosteller, 1951; Rescorla and Wagner, 1972; Sutton and Barto, 1981). In essence, when an individual first encounters a cue followed by an unexpected reward, there is a large discrepancy between what is expected and what actually occurs, producing a large prediction error. However, when an individual learns that a particular cue reliably predicts a motivationally-significant event, there is little error as the discrepancy between what is expected and what actually occurred is diminished. Thus the prediction error functions to drive learning about reward-predictive cues and facilitate more accurate predictions about future rewards.

As the field now stands, phasic activity of midbrain dopamine neurons is considered to represent the prediction error that drives learning as described by the Sutton and Barto (1981) model-free reinforcement learning algorithm. This algorithm explicitly conceptualizes the discrepancies between the expected and delivered outcome as reflecting differences in predicted value, and computes the resultant prediction errors over consecutive time steps during a trial. As a result, the value signal usually produced by reward transfers temporally back to events that reliably precede reward delivery. This effectively endows a cue that predicts reward with the value inherent in the reward itself, rather than just registering when the reward has occurred. In this manner, Sutton and Barto’s (1981) model-free reinforcement learning algorithm explicitly states that the quantitative value inherent in reward transfers back to the antecedent cue predicting its delivery. That is, the predictive cue becomes endowed with the scalar value of the reward rather than explicitly predicting the identity of the outcome which follows cue presentation.

However, thinking about firing from dopaminergic neurons as reflecting a quantitative value signal is limited and does not allow this phasic signal to influence many other complex forms of learning. Firstly, we do not associate all cues with the rewards that they precede. Rather, we select particular cues to learn about on the basis of how well they have predicted that particular reward, or any reward in the past. Such a tendency is encapsulated in models of selective attention in associative theory (Mackintosh, 1975; Pearce and Hall, 1980), where attention directed toward a cue will vary by virtue of its ability to predict reward in the past. But in these models of selective attention, attentional signals are critically influenced by prediction error. That is, the prediction-error signal explicitly informs the change in attention directed toward a cue. Secondly, humans and animals are also capable of inferring associations between cues and rewards in the absence of direct experience. For example, if a cue has been established as predictive of a particular reward and that reward is then devalued outside of the experimental context, the subject will change how they respond to the cue on their next encounter with the cue. This is despite never directly experiencing the now devalued outcome in the presence of the cue. Such learning is typically referred to as ‘model-based’ and is not under the control of the Sutton and Barto (1981) error signal which relies on cached values drawn from direct experiences with cues and outcomes (Dickinson and Balleine, 2002; Berridge, 2012; Dayan and Berridge, 2014). However, recent evidence has begun to suggest that phasic dopamine signals in the midbrain may incorporate model-based information (Bromberg-Martin et al., 2010c; Daw et al., 2011; Hong and Hikosaka, 2011; Aitken et al., 2016; Cone et al., 2016; Sadacca et al., 2016). Such evidence suggests that the dopaminergic error signal may not exist completely apart from these other more complex learning mechanisms.

Here we review empirical studies that challenge and expand on how the dopamine prediction error incorporates and influences learning at associative and circuit levels. In doing so, we will first briefly review the neural correlates of the bidirectional prediction-error signal contained in phasic activity in midbrain dopamine neurons. Then, we will move onto a discussion of how this signal may support a change in attention across multiple attentional systems in distinct brain circuits. Finally, we will review recent evidence that suggests the information contained in the phasic dopamine signal extends beyond that conceptualized by a model-free account. In particular, midbrain dopamine signals appear to reflect information about causal relationships between cues and outcomes in a manner that extends beyond simply encoding the value of a reward predicted by a cue. Such research expands the currently narrow view of how phasic dopamine activity can influence the learning process.

## Reward Prediction Error Signals

At the core of the Sutton and Barto (1981) model-free reinforcement learning algorithm is the concept that prediction error drives learning about cues and the outcomes they predict. That is, if an individual experiences an outcome they did not expect when a cue is presented, a teaching signal will be elicited to update expectations and reduce that prediction error. As a reward in this context is conceptualized as containing an inherent quantitative value, it is this quantitative value that is thought to be transferred to the predictive cue. Effectively, this is argued to endow that predictive cue with the scalar expectation of the upcoming reward. Furthermore, this algorithm proposes that prediction error is bidirectional. Thus, it can drive increases or decreases in learning via signaling a positive or negative prediction error, respectively. A positive prediction error will be elicited when a cue predicts a reward that was more valuable than expected. Here, this signal will act to increase the value attributed to the antecedent cue. However, if an outcome is less valuable than expected on the basis of the expectation elicited by the antecedent cue, a negative prediction error will be elicited and the prediction-error signal will act to reduce the value held by the cue. Essentially, this allows the prediction-error teaching signal to regulate both increases and decreases in the value attributed to predictive cues as a function of the quantitative difference in the reward expected relative to that delivered.

**FIGURE 1:**
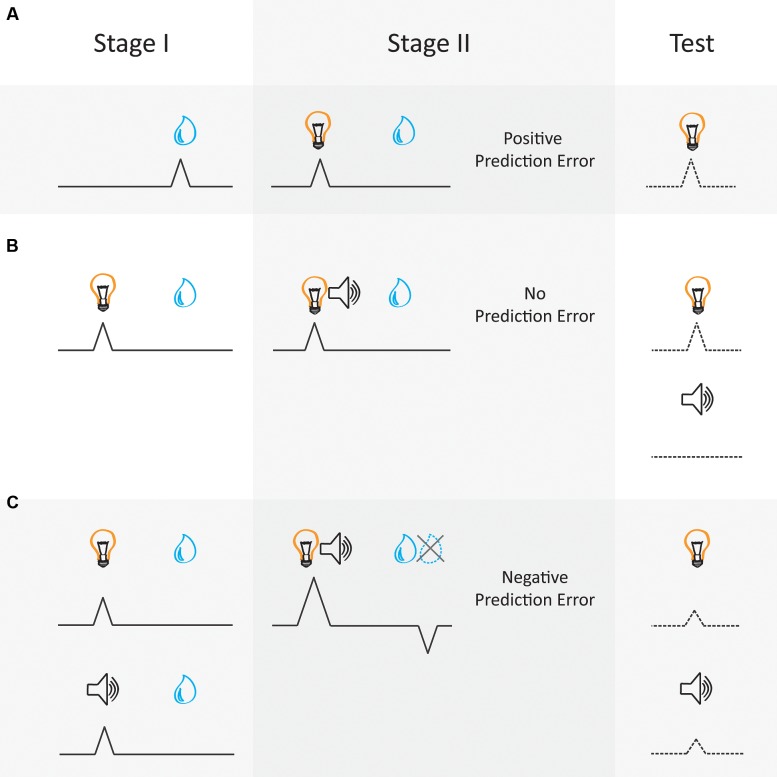
**Dopamine neural correlates follow the laws of prediction error as formalized in Sutton and Barto’s (1981) model-free reinforcement learning algorithm.** According to Sutton and Barto (1981) prediction errors are generated by the quantitative difference between the value of the reward delivered and the value attributed to the reward-predictive cue. Phasic activity of dopaminergic neurons in the VTA can be interpreted as conforming with these predictions. This schematic diagram represents dopamine activity during different Pavlovian conditioning paradigms. Activity is represented by the black lines aligned to a cue (e.g., light or tone, on the left) and reward (e.g., a juice drop, on the right). The dotted lines refer to changes in associative strength. **(A)** Pavlovian conditioning: A reward elicits a positive prediction error when the reward is unexpected, thus an error is made in prediction (Stage I). Dopamine neurons exhibit firing upon the reward delivery. However, with repeated cue-reward pairings this dopamine signal transfers to the reward-predictive cue and diminishes to the reward (Stage II) (Hollerman and Schultz, 1998). As this cue is now predictive of reward, there is a reduction in prediction error at the time of reward and motivated behavior directed toward the predictive cue increases. **(B)** Blocking: a critical aspect of the Sutton and Barto (1981) model is that the learning (or value) about the reward must be shared amongst all present cues. This is referred to in learning theory as a *summed-error term* (Rescorla and Wagner, 1972). This concept is well illustrated by the blocking phenomenon. For example, during Stage I a light cue is trained to predict reward and with training comes to elicit a dopamine signal (Waelti et al., 2001). When a second auditory cue (tone) is presented simultaneously with the light cue and the same quantity of reward is delivered during Stage II, no prediction error is elicited as the reward is already expected and no dopamine signal is exhibited. Behaviorally, learning about the novel tone cue is said to be blocked, and when the cues are presented alone at Test the light cue maintains associative strength but the blocked tone cue does not gain any associative strength. **(C)** Over-expectation: Two different cues (light and tone) that have been separately trained to predict a particular quantity of reward come to each elicit a dopamine prediction-error signal after multiple cue-reward pairings in Stage I. During Stage II, the two cues are then presented as a simultaneous compound, followed by reward given to each trial type during Stage I. This generates a negative prediction error, as the reward is less than the summed expectation of each cue. In this example dopamine signaling is suppressed in response to the over-expected reward not being delivered. This negative prediction error drives a reduction in associative strength so that both cues lose half their associative value when presented alone at Test, assuming these cues are matched for salience (e.g., Chang et al., 2016).

Electrophysiological studies in rodents and non-human primates have demonstrated very convincingly that phasic dopaminergic activity can correlate with the prediction error contained in Sutton and Barto’s (1981) model (**Figure 1**). These neurons show a phasic increase in activity when an unexpected reward is delivered (Ljungberg et al., 1992; Mirenowicz and Schultz, 1994, 1996) or a reward is delivered that was better than expected (Bayer and Glimcher, 2005) (**Figure 1A**). Further, the magnitude of phasic activity correlates with the size of the unexpected reward (Hollerman and Schultz, 1998; Fiorillo et al., 2003; Roesch et al., 2007; Stauffer et al., 2014; Eshel et al., 2016) in a manner that reflects the value of the reward (Lak et al., 2014), value of the future action (Morris et al., 2006) or value of the choice (Roesch et al., 2007), as assessed by the agent’s approach behavior toward to reward-predictive cue. That is, the firing of dopamine neurons changes in response to unexpected rewards or reward-predictive cues in a manner that appears to reflect the subjective value of those rewards. Additionally, the firing of dopaminergic neurons in the midbrain is suppressed when an expected reward is omitted or is worse than expected (Tobler et al., 2003; Brischoux et al., 2009; Matsumoto and Hikosaka, 2009; Lammel et al., 2011, 2014; Cohen et al., 2012). Finally, dopamine neurons also show a slow reduction of firing to the reward over successive cue-reward pairings as the cue comes to reliably predict the reward (Hollerman and Schultz, 1998). That is, the now expected reward elicits minimal phasic excitation when it is presented after the cue, where this activity instead shifts to presentation of the cue itself (see **Figure 1A**). Thus there is a wealth of empirical evidence that can be interpreted as supporting the idea that dopaminergic prediction-error signals comply with those predicted by Sutton and Barto’s (1981) model-free reinforcement learning algorithm.

Another critical aspect of Sutton and Barto’s (1981) model is that associative strength (or value) afforded by the reward must be shared amongst all present cues, referred to as a *summed-error term*. The presence of this summed-error term allowed earlier models (Rescorla and Wagner, 1972) to account for circumstances when cues are presented simultaneously and compete to become associated with the same outcome, as demonstrated in the blocking procedure (see **Figure 1B**). In one example (Waelti et al., 2001), monkeys first received presentations of cue A paired with a juice reward. In the second phase of training, novel cue X was introduced and presented simultaneously with cue A to form a compound AX, where presentation of cue AX was followed with the same juice reward as the first stage of training. During this second phase, monkeys also received a completely novel compound BY followed by the same juice reward. Here, as cue A had already become predictive of reward, there was no error in prediction when compound AX was presented and no associative strength accrued to cue X. On the other hand, as cue BY had never been paired with reward, both cues gained associative strength, sharing the value inherent in the juice reward. Thus when monkeys were tested with cue X and Y they responded more to cue Y as reward was only expected when cue Y was presented. This blocking effect illustrates how prediction error regulates learning by prioritizing cues that have already come to predict reward (Kamin, 1968), allocating less value to a novel cue which does not provide additional information about reward delivery. Thus, prediction errors regulate learning in a manner that produces causal relationships between a cue and the outcome it predicts.

Importantly, midbrain dopaminergic neurons also adhere to the principal of a summed-error term inherent in these models. Specifically, in the blocking design illustrated above (see **Figure 1B**), Waelti et al. (2001) recorded putative dopaminergic neurons during this task. As previously demonstrated dopaminergic neurons increased firing to cue A during the initial phase of training. Then, across the second phase of training dopaminergic neurons maintained similar firing rates to presentations of compound cue AX. Further, dopamine neurons also increased firing rate to the novel compound cue BY across this phase. Critically, in a non-reinforced test where cue X and Y were presented individually without reward, dopaminergic neurons showed robust phasic responses toward cue Y but no response to the blocked cue X, mimicking the behavioral response seen in the blocking paradigm. As cue X and Y were matched for physical salience and paired with an equivalent reward, any difference in the dopaminergic response to these cues could only be attributed to a difference in the summed prediction error, in line with that described by Sutton and Barto (1981).

Until very recently evidence suggesting that phasic activity in midbrain dopamine neurons mimics the scalar prediction error described in Sutton and Barto (1981) has been largely, if not entirely, correlative (Schultz et al., 1997; Roesch et al., 2007; Niv and Schoenbaum, 2008; Iordanova, 2009; Keiflin and Janak, 2015; Holland and Schiffino, 2016; Schultz, 2016). This is because it was difficult to directly manipulate dopamine neurons with the temporal precision and specificity required to directly test this hypothesis. However, the combination of a temporally specific optogenetic approach in addition to the cell type specific transgenic rodent lines has made it easier to manipulate dopamine neurons in a causal manner (Margolis et al., 2006; Lammel et al., 2008; Tsai et al., 2009; Witten et al., 2011; Cohen et al., 2012). This has been hugely advantageous to the study of how prediction-error signals causally influence the learning process. Using transgenic animals expressing Cre recombinase under the control of tyrosine hydroxylase promoter (i.e., Th::Cre lines), a precursor enzyme for dopamine, Cre-dependent viral-vectors injected in to the midbrain can be used to induce expression of the light-sensitive channelrhodopsin-2 (ChR2) or halorhodopsin (NpHR) to selectively activate or inhibit neurons expressing tyrosine hydroxylase (TH+ neurons), respectively. This has afforded neuroscientists the capacity to manipulate dopaminergic neurons in a temporally specific manner that mimics positive or negative prediction errors and assess their causal contribution to the learning process (Steinberg et al., 2013; Chang et al., 2016; Stauffer et al., 2016).

Using this technique, Steinberg et al. (2013) have causally demonstrated that stimulation of dopaminergic neurons in the midbrain can mimic a positive prediction error to drive learning. Steinberg et al. (2013) injected TH-Cre rats with ChR2 in the ventral tegmental area (VTA) and implanted optical fibers aimed at VTA. This allowed phasic stimulation of TH+ neurons in the VTA to mimic the phasic activity typically seen with an unexpected reward and drive excitatory learning. In order to test that these signals do in fact drive learning about reward-predictive cues, they used a blocking procedure, similar to that described above (Waelti et al., 2001; **Figure 1B**). Rats were first presented with cue A that signaled food reward. In a second phase of training, compound cue AX was paired with the same reward. No prediction-error signal should be elicited by the compound cue AX when the reward was presented in the second phase. Therefore, rats would exhibit little learning about cue X as the reward had already been predicted by cue A during training in the first phase of learning. When Steinberg et al. (2013) activated TH+ neurons to artificially mimic a positive prediction error during reward receipt following presentation of the compound cue AX, they found an increase in responding to the usually blocked cue, X, in the subsequent probe test. This result suggests that activating dopaminergic neurons in the VTA mimics a positive prediction error to causally drive learning about the usually blocked cue, X.

If dopamine neurons truly reflect bidirectional prediction errors, it would be expected that briefly silencing their activity would produce a negative prediction error and drive down the ability of a cue to predict reward. In order to determine whether silencing dopaminergic neurons in the VTA could function as negative prediction errors in this manner, Chang et al. (2016) briefly silenced these neurons during a modified version of an over-expectation task (for a simplified illustration see **Figure 1C**). In the standard over-expectation task, the first phase of learning in over-expectation requires that rats learn about two cues (A and B) that independently predict the same magnitude of reward (e.g., one food pellet). During a second phase of learning these two cues are presented as compound AB followed by the same reward. Because cues A and B independently predict the same magnitude of reward, when AB is presented in compound, rats expect delivery of twice the amount of reward (e.g., two food pellets). As rats only receive one food pellet, a negative prediction error is elicited and the associative strength of both cues A and B decreases. However, in a modified version of the over-expectation task, Chang et al. (2016) presented rats with the compound cue AB in the second stage of learning with the expected two food pellets. This change effectively blocks over-expectation from occurring. Against this backdrop, they briefly suppressed TH+ neurons in the VTA during presentation of the reward in AB compound phase of learning. This manipulation decreased the ability of cues A and B to elicit a motivational response in the following probe test, just like what would usually be seen in the traditional over-expectation procedure. Thus Chang et al. (2016) found that transiently suppressing firing of TH+ neurons was sufficient to mimic a negative prediction error. Together, these studies confirm that phasic dopamine can serve as a bidirectional prediction error to causally drive learning.

It is worth briefly noting here that the blocking effect described above has been interpreted as reflecting a performance deficit rather than the result of less learning accruing to the blocked cue X (Miller and Matzel, 1988; Arcediano et al., 2004). According to the comparator hypothesis (Miller and Matzel, 1988), responding to a conditioned cue is in part the result of the strength of the direct association between this cue and the outcome. However, it is also inversely related to the associative strength of any other cue that is presented within a session (i.e., the comparator cue). In this sense, reduced responding to the blocked cue X at test is argued to be the result of increased associative strength that has already accrued to the comparator cue A during the initial phase of conditioning. The evidence in favor of a performance account of blocking is contradictory (Miller and Matzel, 1988; Blaisdell et al., 1999), however, in some instances it has been shown that responding to the blocked cue, X, can be recovered by massive extinction of the comparator cue A which is consistent with the comparative hypothesis (Blaisdell et al., 1999). This research may have consequences for how we interpret VTA DA signals during the blocking task. Specifically, it raises the possibility that the reduced response of dopamine neurons to the blocked cue during the extinction test may reflect the signal used for responding to the blocked cue as predicted by the performance account, rather than the direct association between the blocked cue and the outcome. In this manner, this signal could comprise the quantitative combination of the direct association between the blocked cue X and the outcome, as well as the inverse of the associative strength of the comparator cue A. According to this interpretation, it would not constitute a teaching signal driving learning but rather a signal which reflects this comparative process to produce the reduced response. However, the causal data showing that phasic stimulation of VTA dopamine neurons unblocks learning about the blocked cue X, which results in an increased response to the cue in a subsequent extinction test without stimulation (Steinberg et al., 2013), suggests that these error signals act to causally influence the learning process rather than simply reflecting a comparator signal used for performance.

## Attention

The VTA resides within a rich neural circuit, sending and receiving dense projections from subcortical and cortical regions. Thus it is not surprising that prediction-error signaling in VTA has important and wide-reaching consequences for reward processing across distributed brain reward circuits. For example, prediction-error signaling in VTA influence downstream processing of attention paid toward cues (Corlett et al., 2007; Berridge, 2012; Roesch et al., 2012; Holland and Schiffino, 2016). Interestingly, the manner in which VTA signaling appears to do this has again been predicted by associative models many years before neuroscientists were able to examine these circuits in the way we can today. More interesting still, the mechanisms by which VTA signaling may facilitate attentional processing are diverse and mirrors the controversy in the reinforcement learning literature.

Specifically, a contradiction which has confused undergraduate psychology students for decades is the opposing predictions made by the two dominant attentional theories in associative learning, namely the Mackintosh (1975) and Pearce and Hall (1980) models. On the one hand, Mackintosh’s (1975) model of attention argues that attention will be paid to cues in the environment that are the *best predictors* of a motivationally significant event. Yet, the Pearce and Hall (1980) model of attention predicts the exact opposite- we should attend to cues when we are *uncertain of their consequence*. Indeed, there is strong evidence in humans and other animals for both of these attentional models which suggests that these contradictory attentional processes both exist and in fact contribute to attentional processing.

But each of the attentional strategies proposed by Mackintosh (1975) and Pearce and Hall (1980) models may be beneficial in different circumstances. Consider a situation where we have many cues which predict reward with differing accuracy. Here, it is more efficient to devote attention toward cues that are the best predictors to maximize reward, in line with a Mackintosh (1975) process. However, in a scenario where one or a few cues predict reward it is not always beneficial to devote a lot of attention to a cue that always predicts reward when it is not in direct competition with another cue. Effectively, you do not need to pay a lot of attention to a cue when it is the only one available and attention does not need to bias action selection, in line with the Pearce and Hall (1980) model of attention. Rather, it becomes more important to detect changes in the contingency between a cue and reward to update our knowledge of these relationships.

Evidence for a view where different scenarios recruit different attentional processes is supported by the fact that findings consistent with either Mackintosh (1975) or Pearce and Hall (1980) models tend to be found using different experimental parameters. Individuals are generally found to attend to the best predictors of reward when parameters promote high cue competition (Mackintosh, 1965, 1973, 1976; McLaren and Mackintosh, 2002; Le Pelley et al., 2011, 2013) whereas effects suggesting individuals attend more to inconsistent predictors are generally found in cases where one or few cues are available (Hall and Pearce, 1979; Wilson et al., 1992; Griffiths et al., 2011; Esber et al., 2012). In fact, recent models of associative learning have formalized this concept to predict how attention will change across learning under these different circumstances, via hybrid models (LePelley and McLaren, 2004; Pearce and Mackintosh, 2010) or models that reconcile the roles of predictiveness and uncertainty (Esber and Haselgrove, 2011).

Important to the current discussion is that models of reinforcement learning utilize prediction errors in two ways (Pearce and Hall, 1980; Mackintosh, 1975). Firstly, prediction-error signaling regulates the amount of learning that can occur on any single cue-reward pairing. That is, the magnitude of the difference between the expected and experienced reward will determine how much learning can accrue to the cue in subsequent trials. However, prediction errors are also argued to regulate the change in attention devoted to that cue, which will dictate the rate of learning and, therefore, which cues are learnt about. In Mackintosh’s (1975) model, attention declines to cues that result in larger prediction errors and are, therefore, poor predictors of reward. Here, attention increases toward cues which results in a smaller prediction error relative to other present cues. In direct contrast, the Pearce and Hall (1980) model posits that attention is maintained to a cue that produces larger prediction errors. According to Pearce and Hall (1980), attention decreases when prediction errors are small, consequently well-established predictors will receive less attention.

**FIGURE 2:**
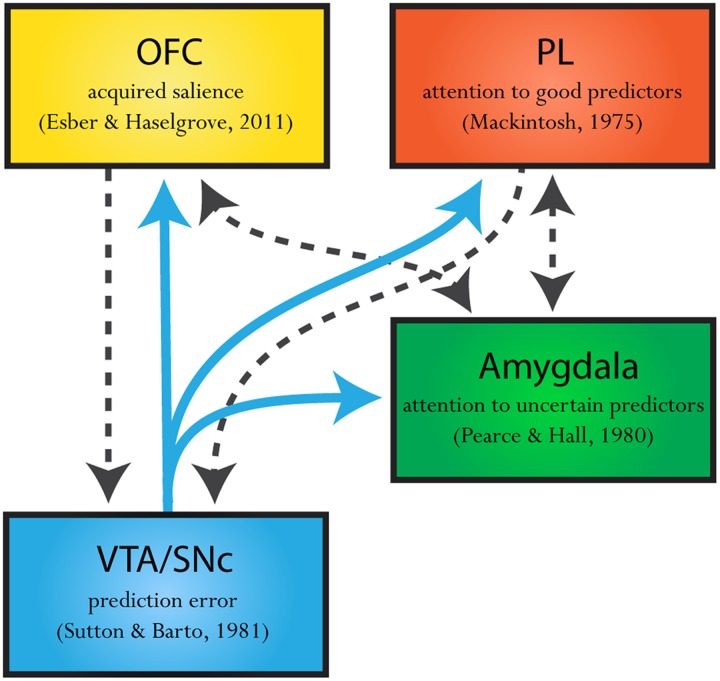
**Theorized neural circuitry of a dopaminergic prediction error projections informing attentional processes.** Dopamine prediction-error signaling (blue solid arrows) regulates the amount of learning that can occur on any one cue-reward pairing. The midbrain, ventral tegmental area and substantia nigra (VTA/SNc) sends out dense dopaminergic projections to the amygdala, prelimbic cortex (PL) and orbitofrontal cortex (OFC). The amygdala, PL, and OFC have all been implicated in distinct attentional processes. Specifically, the amygdala has been implicated in attending toward cues which inconsistently predict an outcome, uncertain predictors, in line with a Pearce and Hall (1980) mechanism. On the other hand, the PL region of the mPFC has been implicated in promoting attention toward cues which are good predictors of an outcome relative to other reward-predictive cues, as predicted by Mackintosh’s (1975) model of attention. Further still, neural correlates in the OFC appear to respond to cues which are good and uncertain predictors, where activity in a proportion of these neurons is high for cues that are consistent predictors of reward yet higher still for cues that are inconsistent predictors of reward (Ogawa et al., 2013) as predicted by Esber and Haselgrove (2011). Suggesting a role for the OFC in modulating acquired salience. The commonality of these three distinct attentional theories is that they all propose that prediction errors influence how much attention will be paid toward a cue. The presence of this neural circuit (illustrated above), where the VTA/SNc sends distinct projections to these three attentional regions provides a plausible circuit whereby prediction errors could influence attentional processing in different ways. Furthermore the PL, OFC, and amygdala are interconnected in such ways that these regions may integrate attentional information as described in two-process models (black dashed arrows; see McDonald, 1987, 1991; Vázquez-Borsetti et al., 2009).

The neural evidence also favors the presence of both these dissociable attentional processes. Specifically, evidence suggests that a Mackintosh-like (Mackintosh, 1975) attentional process occurs in the prelimbic cortex (PL) in the medial prefrontal cortex (mPFC) (Sharpe and Killcross, 2014, 2015), while neural activity in basolateral complex of the amygdala (BLA) reflects a Pearce and Hall (1980) signal (Roesch et al., 2010, 2012; Esber et al., 2012; Esber and Holland, 2014). Of course, such opposing attentional processes do not exist in isolation. It is well-established that VTA sends out dense projections to both the PL and BLA, providing a plausible circuit through which prediction-error signaling could influence attentional signals in these regions (see **Figure 2**). The presence of these dissociable neural circuits strengthens recent attempts to build models of associative learning which allows prediction error to influence attentional processing in these different ways (LePelley and McLaren, 2004; Pearce and Mackintosh, 2010; Esber and Haselgrove, 2011). That is, the neural evidence supports the idea that prediction error can regulate not only the amount of learning available on any one trial but also to influence different types of attentional processing in distinct circuits. In this section, we will examine the neural evidence for each of these systems alone and will then review recent attempts at a reconciliation between these attentional processes.

As a brief note here, we would acknowledge that we have focused on reviewing the literature which conceptualizes attention as a modulator of learning rates. That is, we have focused on models in which attention directly acts to regulate the amount of learning that is attributed toward a particular cue on any one trial. Conceptualizing attention in this manner has become common place within the associative learning literature, predominantly driven by studies utilizing rodents (but see: Le Pelley et al., 2011, 2016). However, there is a wealth of literature on attention which conceptualizes attention in other ways, mainly driven by studies in humans and non-human primates. For example, attention may also be conceptualized as modulating the bottom-up sensory processing of cues, or as influencing activation of cue-response associations (to name just a few; Miller and Cohen, 2001; Hickey et al., 2006). These mechanisms focus on how cues are processed relative to other present cues or how cues can influence the ability to elicit an associated response, but not the ultimate amount of learning that accrues to the cue itself. While the relationship between attention and behavior is likely the same across both sets of definitions- where increases in attention act to increase behavior directed toward a cue, and decreases in attention the reverse- there are significant differences in *how* attention is hypothesized to influence learning and/or behavior. Given this, it is likely that future integration of these fields would likely be fruitful in understanding attentional processing across species (see e.g., Hickey et al., 2006, 2011, 2015; Jovancevic et al., 2006; Hare et al., 2011; Hickey and Theeuwes, 2011; Lim et al., 2011; Gottlieb, 2012; Gottlieb et al., 2014; Theeuwes, 2013; Tommasi et al., 2015; Wilschut et al., 2015 for a more comprehensive review on these attentional theories).

### Pearce and Hall (1980) Model of Attention

A sub-nucleus of the amygdala complex, the BLA, is a region that receives extensive dopaminergic input from midbrain dopamine neurons (Swanson, 1982) and shows increases in neural activity when an unexpected event occurs whether it is rewarding or aversive (Belova et al., 2007, 2008; Herry et al., 2007; Roesch et al., 2010; Tye et al., 2010; Li et al., 2011; Beyeler et al., 2016). Notably these signals seem to conform closely to what is predicted for a Pearce and Hall (1980) attentional signal. Specifically, Roesch et al. (2010) recorded neurons in the BLA during a task in which expectations were repeatedly violated. Here, rats were trained to enter a food well after two odors were presented. One of these odors predicted that the right well would be reinforced and the other predicted that the left well would be reinforced. At the beginning of each training block, the timing and size of rewards delivered in these wells were manipulated to either increase or decrease the value of the reward delivered at each well. Roesch et al. (2010) found that a population of neurons in the BLA responded similarly to both upshifts and downshifts of reward value. Specifically, these neurons increased their firing rate when expectations were violated, regardless of whether they constituted decreases or increases in reward value. This *unsigned* or unidirectional error signals are reminiscent of that described by the Pearce and Hall (1980) model of attention, whereby attention is enhanced by means of an absolute value prediction error. In line with an attentional interpretation, this neural signal was integrated across trials and correlated with greater levels of orienting toward the predictive cues after changes in reward, where orienting constitutes a reliable measure of overt attention in the associative learning literature. Functional inactivation of the BLA disrupted changes in orienting behavior and reduced learning to respond to changes in the reward. The findings from this study suggested that the BLA is critical in driving attention for learning according to a Pearce and Hall (1980) mechanism.

Notably, Esber et al. (2012) further demonstrated that the ability of BLA neurons to exhibit this Pearce-Hall signal is dependent on dopaminergic input from the VTA. Specifically, Esber et al. (2012) recorded neurons in the BLA of rats with ipsilateral sham or 6-hydroxydopamine (6-OHDA) lesions of the VTA during the choice task described above (Roesch et al., 2010). They found that neurons in the BLA of intact rats again showed this characteristic increase in activity to either upshift or downshifts in reward value in this task. However, BLA neurons in 6-OHDA-lesioned rats failed to show this attentional signal. Interestingly, despite the deficit in attentional signaling, neurons of lesioned rats still exhibited a sensitivity to value *per se*. That is, neurons in the BLA of lesioned rats continued to respond more to cues predicting high magnitude of reward and less to those predicting lower amounts of reward. This demonstrated that dopaminergic activity in the VTA is necessary for neurons in the BLA to exhibit this unsigned prediction error but not for the ability of this region to encode other characteristic neuronal signals. Of course, while 6-OHDA lesions suppress phasic dopamine signaling, these lesions also suppress tonic dopamine signaling in the VTA. Thus, while it is clear that dopaminergic input appears to be necessary for neurons in the BLA to exhibit unsigned attentional signal in a manner described by the Pearce and Hall (1980) model, future research is necessary to confirm that relay of phasic VTA DA prediction-error signals produce an increase in attention toward a cue when expectations have been violated.

Interestingly, the central nucleus of the amygdala (CeA), another sub-nucleus of the amygdala complex, has also been implicated in attentional processes predicted by the Pearce and Hall (1980) model. Using a serial-conditioning task designed by Wilson et al. (1992), lesions of the CeA disrupted surprised-induced increments in attention (Holland and Gallagher, 1993). Here, two cues were presented as a serial compound, whereby a light consistently predicted presentation of a tone. On half of the trials, the serial compound was followed by reward. According to the Pearce and Hall (1980) model, as the light consistently predicted the tone, the attention to the light should be low. In a second phase, one group of rats continued this training. However, another group now only received the light prior to the tone on reinforced trials. On non-reinforced trials, the light was presented alone. That is, in this second group the light no longer consistently predicted the tone. According the Pearce and Hall (1980) this surprising omission of the tone should increase attention paid to light. Consistent with this prediction, sham-lesioned rats who received the surprising omission of the tone later showed faster acquisition of responding to the light when it was paired with a novel outcome than sham-lesioned rats that had consistent training. This showed that attention to the light increased as a consequence of the omission of the tone which facilitated later learning about the light. However, rats with lesions of the CeA failed to show this faster rate of learning as a consequence of the surprising omission of the tone. This demonstrated that the CeA is necessary for surprise-induced increments in attention, in line with predictions made by the Pearce and Hall (1980) model of attention.

The role for the CeA in surprise-induced increments in attention is not dissimilar from the attentional role attributed to the BLA. That is, both regions have been implicated in increases in attention as a result of the violation of expectancies in line with the Pearce and Hall (1980) model. However, while this attentional process in BLA appears to be the product of direct dopaminergic projections from the VTA, the CeA does not receive this input (Pitkanen et al., 2000). Rather, the CeA receives projections from the substantia nigra (SNc) that appears to facilitate this attentional process. Specifically, Holland and Gallagher (1993) demonstrated that disconnection of the SNc and CeA using ibotenic acid lesions of CeA in one hemisphere and 6-OHDA lesions of SNc in the opposite hemisphere prevented increasing attention to the light cue when it no longer consistently predicted the tone in the serial-conditioning task described above (Lee et al., 2006). This demonstrates that it is dopaminergic input from the SNc that facilitates attentional processing in the CeA, rather than from the VTA, as appears to be the case in the BLA. This anatomical difference invites the possibility that the attentional processes taking place in these regions are fundamentally different. This possibility is supported by the finding that lesions of the CeA also interfere with the basic acquisition of a conditioned orienting response to a reward-predictive cue, whereas BLA lesions do not (Holland and Gallagher, 1993, 1999). This has led to the argument that CeA drives behavioral changes resulting from changes in attention (Holland and Gallagher, 1999; Holland et al., 2001). Thus, dopamine projections from the SNc to CeA may function to produce overt behavioral changes in attention to influence rates of learning rather than modulating the rate at which a cue becomes associated with an outcome *per se*, which may be a point of difference from attentional processing which takes place in the BLA.

### Mackintosh (1975) Model of Attention

In contrast to the role of the CeA and BLA in an attentional process implicated in the Pearce and Hall (1980) model, inhibition of activity in the rodent mPFC has been causally demonstrated to produce deficits in modulating attention toward cues in a manner akin to that described by Mackintosh’s (1975) theory of attention (Sharpe and Killcross, 2014, 2015). As would be expected from a region modulating attention according to a Mackintosh (1975) attentional process, lesions or inactivation of the mPFC produce deficits in tasks that promote high competition between multiple cues. The classic finding is that mPFC lesions produce impairments in extradimensional set shifting, where subjects have to attend toward a set of cues that are established as predictive of reward and disregard other present, but irrelevant, cues (Birrell and Brown, 2000). Such effects have more recently been attributed to the PL region of the mPFC, where a role for this region in attention can now be explicitly dissociated from a role in error correction (Sharpe and Killcross, 2014, 2015). For example, PL lesions do not disrupt expression of the blocking effect but selectively impair the ability to stop attending toward the redundant blocked cue (Sharpe and Killcross, 2014). Here, rats received PL lesions prior to a typical blocking paradigm. In stage I of this task, rats received pairings of cue A with reward. In stage II cue A was paired with novel cue B and the same magnitude of reward. In this same stage, rats were also presented with a novel compound CD and the same reward. PL lesions did not affect blocking to cue B relative to cue D, demonstrating an intact error-correction process dependent on prediction-error signaling in the VTA. However, after the blocking procedure these same animals were presented with the blocked cue B and then presented with reward. In line with a Mackintosh attentional process, sham-lesioned rats demonstrated slow learning about cue B, suggesting attention had declined toward this cue as it was previously a poor predictor of the outcome. However, rats with PL lesions exhibited faster learning about this cue suggesting they had not down-regulated attention toward blocked cue B. This demonstrates that the PL cortex is necessary to direct a preferential degree of attention toward predictive cues while not being necessary to allow learning to be regulated by prediction error *per se*.

Interestingly, VTA sends a particularly dense projection to the PL region (Bentivoglio and Morelli, 2005; Björklund and Dunnett, 2007). While the causal influence of these signals on attentional processing are lacking and constitute an interesting direction for future research, there is considerable evidence that phasic firing in VTA dopamine neurons directly affects neurons in the mPFC (Niki and Watanabe, 1979; Tzschentke and Schmidt, 2000; Rushworth et al., 2011). For example, electrophysiological studies have demonstrated that burst stimulation of VTA promotes prolonged depolarization of mPFC pyramidal neurons, constituting a change to an ‘up state’ where the membrane potential of neurons in this area is brought close to firing threshold (Lewis and O’Donnell, 2000). Such research may suggest that phasic firing in VTA may act to enable plasticity in mPFC circuits, where firing rates tune to cues which are good predictors of an outcome (Gruber et al., 2010). In line with this, evidence from electrophysiology (Niki and Watanabe, 1979) and functional magnetic resonance imaging (fMRI) studies (Rushworth et al., 2011), have shown that activity in mPFC encodes both the value of the upcoming rewards predicted by cue presentation as well as a depression in activity during the omission of an expected reward. This is distinct from activity seen in the BLA which, as discussed above, exhibits a general increase in firing in response to both delivery and omission of expected reward. While the mechanism by which burst firing in VTA dopamine neurons influence attentional processing in PL cortex remains to be clarified, the ability of phasic responses to influence activity in PL cortex suggest that prediction errors in VTA may influence activity in the PL cortex to produce an attentional signal in line with that predicted by Mackintosh’s (1975) model of attention and dissociable from that seen in other regions of the brain.

It is worth noting here that the neural signal predicted by Mackintosh’s (1975) model of selective attention is not as simple as an increase in responding to cues which are predictive of reinforcement. Indeed, many regions of the brain show increases in activity to predictive cues. The uniqueness of Mackintosh’s (1975) predicted attentional signal is perhaps best illustrated by the model’s predictions in times of cue competition. Take, for example, the overshadowing paradigm, whereby an audio–visual compound is presented with reward. If this compound cue differs in intrinsic salience, after the first few trials associative strength will decrease toward the less intrinsically salient element of the compound (a dim visual cue) as the more intrinsically salient element of the compound (a loud auditory cue) accrues associative strength more quickly, and this overshadows the less intrinsically salient element. Unlike most models of reinforcement learning (Rescorla and Wagner, 1972; Pearce and Hall, 1980; Sutton and Barto, 1981), Mackintosh’s (1975) model does not use the summed-error term developed in the Rescorla and Wagner (1972) model, later adapted by Sutton and Barto (1981). Instead, learning to predict an outcome need not be shared by all present cues. Mackintosh’s (1975) model uses attentional change to explain the decrement in learning when multiple cues of different intrinsic salience predict the same outcome. More formally, the change in a cue’s associative strength is based on that individual cue’s prediction error. Thus the less intrinsically salient cue is learnt about more slowly and is, therefore, a less reliable predictor of reward and learning about this cue stops. In line with a role for the PL cortex in a Mackintosh (1975) attentional process, inactivation of the PL cortex specifically impairs overshadowing of the less intrinsically salient visual cue paired with a shock in a procedure that promotes this form of overshadowing (Sharpe and Killcross, 2015).

The presence of an individual-error term in the Mackintosh (1975) model has consequences for the nature of the attentional signal that may expected in neural regions contributing to this attentional process. Specifically, Mackintosh’s (1975) model would predict high attention across the first few trials of overshadowing to both elements of the compound, with a selective decrease to the visual element of the compound. This is despite a relative increase in associative strength attributed to the visual cue from the start of conditioning. Overshadowing of one element of the compound is not predicted by models that utilize a summed-error term (Rescorla and Wagner, 1972; Sutton and Barto, 1981). Rather, models using a summed-error term would predict mutual overshadowing to both elements of the compound. That is, both the salient auditory and less salient visual cue will accrue less associative strength than they would if conditioned individually by virtue of sharing the learning supported by the reward (though the degree to which this occurs is dependent on intrinsic salience). Further, these models are not attentional in nature and would therefore not predict that the signal to either element of the compound would decrease across learning. Thus a search for a Mackintosh (1975) neural signal would have to take into account the complexities of the model rather than just looking for an increase in activity toward predictive cues.

### Unifying Models of Attention: Esber and Haselgrove (2011)

So far we have reviewed evidence for each attentional process (Mackintosh, 1975; Pearce and Hall, 1980) as potentially independent yet interactive processes, in line with several hybrid or two-process models of attention (LePelley and McLaren, 2004; Pearce and Mackintosh, 2010). However, another model attempts to reconcile these processes into one mechanism in which attention is directed by *both* predictiveness and uncertainty (Esber and Haselgrove, 2011). Unlike attentional models where the size of the prediction error regulates the amount of attention paid to a cue (Mackintosh, 1975; Pearce and Hall, 1980), the Esber and Haselgrove (2011) model assumes that *acquired salience* of a cue will change with how well it predicts an outcome. At first glance, this sounds similar to Mackintosh’s (1975) model of attention. Humans and animals attend to good predictors of reward. However, the Esber and Haselgrove (2011) model also predicts that the omission of an expected reward can function as an effective reinforcer. This is because the frustration caused by omission of an expected reward is also a motivationally-potent event. Thus, a cue that probabilistically predicts both delivery and omission of expected reward will have increased acquired salience relative to a cue that consistently predicts reward or omission alone, as the former now becomes predictive of two outcomes. Thus, this theory can account for evidence suggesting that humans and animals attend toward good predictors of an outcome (Mackintosh, 1975) while also maintaining attention toward cues which are uncertain predictors of an outcome (Pearce and Hall, 1980).

The critical assumption here is that a cue that is partially reinforced will acquire higher salience relative to a cue that is consistently rewarded (Esber and Haselgrove, 2011). Recently, evidence has emerged showing that some neurons in the orbital frontal cortex (OFC) show such a pattern of responding in anticipation of reward following cue presentation (Ogawa et al., 2013). Most notably, in this study, rats were presented with four odor cues. Two cues consistently predicted reward (100%) or no reward (0%), and two cues inconsistently predicted reward (67%, 33%). Here, around half of the reward-anticipatory neurons in OFC exhibited their highest responding when cues inconsistently predicted reward (67%, 33%). However, critically, these neurons also showed higher firing to certain reward (100%) than certain non-reward (0%), which was near baseline. This pattern- baseline firing in anticipation of non-reward, and increased firing in anticipation of certain reward, and still higher firing to uncertain reward- was perfectly in line with the predictions of the Esber and Haselgrove (2011) model. Future research should explore whether the attentional signal described by Esber and Haselgrove (2011) is pervasive across other systems implicated in attention which may help to reconcile the apparent contradiction in the associative world without appealing to a two-process model. If this is not the case, there are connections between the PL, OFC, and BLA (McDonald, 1987, 1991; Vázquez-Borsetti et al., 2009) that may allow integration of multiple competing processes (see **Figure 2**).

## More Complex Associative Models

The research above describes how prediction errors may regulate both the rate and amount of learning attributed to a reward-predictive cue across several dissociable circuits. But this is only half the story. Our experience with cues in the environment is often more complex than a discrete cue predicting a rewarding outcome. For one, our experiences are often different depending on context. Consider a veteran coming back from war. During their time at war, they probably formed a strong association between loud noises and negative consequences. However, when the veteran returns home it is far more likely the case that a loud noise signals something innocuous like a slamming door or misfiring engine. It is important in these circumstances that an individual has learned (and can recall) context-specific associations, and does not generalize negative experiences into neutral contexts (Rougemont-Bücking et al., 2011; VanElzakker et al., 2014; Sharpe et al., 2015).

Interestingly, dopamine neurons in the VTA can exhibit context-specific prediction errors that reflect context-specific learning (Nakahara et al., 2004; Kobayashi and Schultz, 2014). For example, Nakahara et al. (2004) trained monkeys to expect reward when presented with a visual cue. Here, one group of monkeys experienced one set of contingencies (a context-independent task), and another group were given another set of contingencies (the context-dependent task). In the ‘context-independent’ version of the task, the cues were presented with reward 50% of the time, where reward was delivered according to a random distribution. In the ‘context-dependent’ version of the task, the cues were also reinforced 50% of the time, however, the rate of reinforcement changed depending on the previous run of reinforcement. Here, if monkeys had experienced a long run of non-reinforcement across six trials, they were guaranteed reward on the next trial. So unlike monkeys in the context-independent task, monkeys in the context-dependent task should be able to learn when to expect a rewarded trial. If prediction errors can encode context-dependent information then dopamine activity on the guaranteed rewarded trial after a run of six loses should be minimal, despite the trial constituting an increase in the magnitude of reward that would usually elicit a large prediction error. Sure enough, with extended training prediction errors adjusted to the contextual rule and were modified depending on the prior history of reward. That is, prediction-error signaling was low on trials where monkeys anticipated reward after a long run of unrewarded trials but high when unexpected reward was given before this run of six loses was over. This demonstrates that VTA dopamine prediction-error signals are capable of reflecting information garnered from complex scenarios (Bromberg-Martin et al., 2010a; Takahashi et al., 2011). Since then, it has also been demonstrated that prediction errors can also be modulated by visual background cues (Kobayashi and Schultz, 2014), showing that prediction errors can adjust to both implicit and explicit contextual cues.

Such a finding is compatible with Sutton and Barto’s (1981) model-free reinforcement algorithm. This is because this theoretical account relies on the concept of *state*. Here, state is defined as any array of salient observations, either explicit or implicit, that is associated with a particular prediction about the value of upcoming rewards. Hence, during conditioning when a subject experiences presentation of a cue which has been established as predictive of reward, the cue state accrues the value inherent in the reward. Thus, delivery of the reward at the end of cue presentation will not be surprising and a prediction error will not be signaled. Further, the concept of state need not be defined only by reference to the temporally predictive cue. Rather, it can encompass many attributes of the trial. For example, it could include information about how long it has been since reinforcement or other sensory cues (e.g., contextual cues) available on that trial (Nakahara et al., 2004; Redish et al., 2007; Gershman et al., 2010; Nakahara and Hikosaka, 2012; Nakahara, 2014), basically anything that has been directly experienced as associated with reward in the past. Thus, the finding that VTA dopamine prediction-error signals adjust with either implicit or explicit contextual cues can be easily explained within the traditional view that the dopamine error system emits a signal synonymous with that predicted by model-free algorithms such as that described in Sutton and Barto (1981). This is because different expected values can be assigned to a particular state that are capable of containing information beyond the discrete cue that predicts reward (Bromberg-Martin et al., 2010c; Hong and Hikosaka, 2011; Aitken et al., 2016; Cone et al., 2016).

Not only are dopamine prediction errors capable of reflecting state-specific associations, dopamine prediction errors are also theorized to contribute to the creation of new states which allow for the development of state-specific associations (Gershman et al., 2010; Gershman et al., 2013). Specifically, it is thought that persistently large prediction-error signals may serve as a segmentation signal that alerts the individual to a new state of the world and to form a state-specific association. Take for example, the context-specificity of extinction learning. If a predictive cue is suddenly presented without its predicted outcome, humans and other animals do not unlearn the original cue-outcome association. Rather, they will attribute the change in contingency to any perceived change in the experimental circumstance (Bouton, 2004). Thus, responding to the predictive cue will re-emerge when the experimental circumstance no longer reflects that present in extinction (e.g., the passage of time or a physical change in context; Bouton, 2004). According to Gershman et al. (2010), the large prediction errors present at the beginning of extinction leads an individual to infer a new state and form a context-dependent association specific to the extinction context. In line with this theory, Gershman et al. (2013) have shown that using a gradual extinction procedure, where prediction errors during extinction were reduced by sporadically presenting reinforced trials, reduced the recovery of responding to the predictive cue following the passage of time. This is consistent with an idea that experimentally manipulating the degree of prediction error during extinction reduced the likelihood that a subject will infer a new state and form a context-specific association.

Of course, learning also often extends beyond a reaction to explicit and implicit sensory cues. Humans and other animals are capable of constructing rich associative models of the world which can be flexibly utilized in the absence of direct experience. In such models, a behavioral choice is often made by simulating all possible consequences and selecting the response that is associated with the outcome that is most favorable to the participant. The construction of such models is typically referred to as ‘model-based’ learning and contains information about value as well as the identity of cues, responses, and rewards. Such learning is typically considered to be independent of a dopaminergic prediction-error system under current interpretations of these signals (Schultz, 1997, 2002, 2007). However, recently research has begun to emerge which suggests that dopaminergic prediction errors may contain model-based information (Daw et al., 2011; Sadacca et al., 2016). For example, dopaminergic prediction errors are influenced by OFC activity, known to be involved in model based behaviors (Takahashi et al., 2011). Further, Daw et al. (2011) recently found evidence for information consistent with a model-based account of behavior in the ventral striatum, traditionally thought to receive a model-free prediction error from VTA dopamine neurons (Suaud-Chagny et al., 1992; Day et al., 2007). Here, they tested human participants on a two-stage decision task. In the first stage, subjects are presented with two pictorial cues. A choice of one cue would lead to a second stage where another set of cues (set 1) are presented the majority of the time, where the choice of the other would lead to a different set of cues (set 2) being presented most of the time. In this second stage, choice of one of the pictorial cues in the two different sets leads to either low or high monetary reward. On rare transitions, the first-stage choice of the set 1 cues would lead to the set 2 of pictorial cues that it is not usually associated with that first-stage choice. The reasoning here is that if the rare transition to the set 2 cues ended up with a choice that lead to an upshift in monetary reinforcement, a model-based agent would select the choice in the first-stage that most likely produces the set 2 cues. That is, they would actually produce a different response from the last reinforced response as it is more often that the alternate choice led to presentation of the set 2 cues. However, a ‘model-free’ agent, would make the same choice as the last trial. This is because the response on the last trial has just been reinforced and value of that action updated. In line with a model-based account of this behavior, when participants had been reinforced after the rare transition, they choose the different response on first-stage of the next trial that was likely to lead to the pictorial cues that signals greater reinforcement. Further, the Blood Oxygenation Level Dependent (BOLD) activity of this model-based choice were specifically found in ventral striatum, where activity tracked individual differences in degree of model-based behavior. This challenges the traditional assumption that such activity reflects a model-free error signal from VTA dopamine, suggesting this signal facilitates the use of more complex choice behavior that requires an associative structure of the task.

In further support of this notion, Sadacca et al. (2016) have recently found direct evidence that VTA dopamine phasic signals in the rodent encodes model-based information. Using a sensory-preconditioning task, Sadacca et al. (2016) found that VTA dopamine neurons emit their traditional phasic signal toward a cue that has not been directly paired with reward but, rather, has come to predict reward via its associative relationship with another reward-paired cue. Sensory preconditioning involves first pairing two neutral cues as a serial compound in the absence of any reward. Following this preconditioning phase, one of these cues is then paired directly with reward during conditioning. As a consequence of this training, both the reward-paired and neutral cue will now elicit the expectation of reward. Thus, the cue not directly paired with reward also acquires an ability to predict reward via its prior association with the to-be-conditioned cue. Such a prediction is model-based as updating learning in the absence of direct experience requires the existence of a mental map of relationships between cues that can be flexibly adapted to incorporate the new information. Interestingly, Sadacca et al. (2016) found that VTA dopamine neurons responded to both the cue directly paired with reward and the neutral cue that came to predict reward by virtue its associative link with the reward-paired cue in the preconditioning phase. This data clearly demonstrates that VTA dopamine neurons encode associations that reflect model-based inference not based on direct experience. Thus emerging evidence from both the human and rodent literature has begun to suggest that the dopaminergic prediction-error system contains information that goes above and beyond that appropriately described as a model-free value signal described in Sutton and Barto (1981).

## Outstanding Questions

### How Does the Diversity of VTA Dopamine Neurons and Their Projection Targets Lend to Our Understanding of How Associative Learning Systems Interact?

A growing interest in the field is the investigation of the heterogeneity of dopaminergic neurons in the VTA and the diversity of their neurons targets (Lammel et al., 2011; Parker et al., 2016; Morales and Margolis, 2017). For example, studies have identified that distinct populations of dopamine neurons in the VTA that show preferential increases in firing to either rewarding and aversive outcomes and the cues which predict their occurrence (Matsumoto and Hikosaka, 2009; Bromberg-Martin et al., 2010b). In parallel, research has shown that distinct populations of VTA dopamine neurons receive input from the laterodorsal tegmentum (LDT) and lateral habenula (LHb), argued to underlie these appetitive and aversive responses, respectively (Lammel et al., 2012). These inputs from LDT and LHb synapse preferentially on VTA dopamine neurons projecting to nucleus accumbens (NAc) and PFC, respectively. These studies are a few of a host of studies which are beginning to identify disparate populations of VTA dopamine neurons that appear to show distinct and complex interactions with wider neuronal systems where they contribute to behavior in diverse ways (Matsumoto and Hikosaka, 2007; Jhou et al., 2009; Lammel et al., 2011; Eban-Rothschild et al., 2016; Parker et al., 2016). Additional complexity of the VTA dopamine system comes from recent evidence which suggests that VTA dopmaine neurons also release other neurotransmitters such as glutamate and GABA. Thus this emerging research begins to paint a complex picture of how VTA dopamine neurons may contribute to learning and behavior which may continue to challenge a perception of the prediction error as a cached-value signal. The continuation of such research will undoubtedly shed light on the ways in which VTA dopamine prediction-error signaling contributes to attentional and model-based learning described in this review.

### What about Non-dopaminergic VTA Neurons?

**FIGURE 3:**
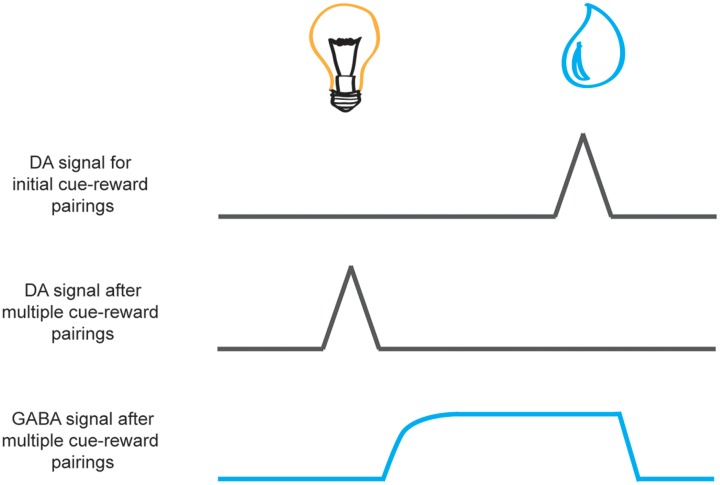
**Schematic of dopamine and GABA reward prediction-error activity during learning.** Neural activity is aligned to cue presentation (e.g., light, on the left) and reward presentation (e.g., a drop of juice, on the right). While phasic activity of dopamine neurons (black lines) are elicited by unexpected reward delivery upon initial cue-reward pairings (top) with repeated cue-reward pairings the signal at the time of reward receipt wanes as the reward becomes predicted by the cue (middle). This transition occurs gradually over successive trials in accordance with traditional learning models of prediction error (Rescorla and Wagner, 1972; Sutton and Barto, 1981). It is speculated that this reduction in the dopamine signal to the reward may result from inhibition of dopamine neurons by GABAergic neurons in the VTA (bottom, blue line) that is initiated after cue offset and persists during reward delivery (Houk et al., 1995; Cohen et al., 2012).

The prediction-error signal to the reward wanes across successive cue-reward parings as the cue comes to reliably predict the reward. However, with this decrease in signal at the time of the reward, we also see an increase of dopamine signaling to the reward-predictive cue. This phasic response to the cue is thought to reflect the cached value inherent in the reward it predicts. It has been suggested that this reduction in the neural response at the time of reward, as a result of expectation elicited by a cue, may arise from inhibition of dopamine neurons that is initiated after cue offset and persists during reward (see **Figure 3**). GABAergic neurons in the VTA are one possible candidate proposed to provide this inhibitory signal. Recently, Cohen et al. (2012) recorded GABAergic neurons in animals well trained on a simple cue-reward procedure where different odor cues predicted either big reward, small reward, nothing, or punishment. Cohen et al. (2012) found that dopaminergic neurons responded to cues in a manner consistent with the quantitative value it predicted. However, while GABAergic neurons were excited by predictive cues, they exhibited sustained activity across the delay between the cue and the expected reward (see **Figure 3**). The authors concluded that this signal from GABAergic neurons counteracts the excitatory drive of dopaminergic neurons when a reward has been predicted to ensure that a prediction-error signal is not elicited when an expected reward is delivered. Thus, these GABAergic neurons may contribute to the development of a reduction in the dopaminergic response during reward receipt as the cue comes to predict the reward. Future studies may continue to investigate the causal role of these neurons in learning and to determine which inputs from other regions provide the expectancy signal to allow GABAergic neurons to modulate dopaminergic prediction-error signals in the VTA.

### Is Learning Always Distributed in Accordance with a Summed-Error Term?

As it stands we have argued that dopamine signaling in the VTA can support learning in a manner that is consistent with multiple theories of associative learning. In doing so, we have predominantly focused on how VTA dopamine may relay a summed-error term to facilitate cue processing in other brain regions (Rescorla and Wagner, 1972; Sutton and Barto, 1998). However, empirical data has shown that learning can also be governed by an individual-error term; as such learning on any one trial need not be equally distributed across cues present on a trial even if they are of equal salience (Le Pelley and McLaren, 2001; LePelley and McLaren, 2004; Leung and Westbrook, 2008). One of the most convincing findings in favor of the presence of individual-error terms comes from studies of causal learning in humans (LePelley and McLaren, 2004). Specifically, Le Pelley and McLaren (2001) looked at the distribution of associative change between the elements of a compound composed of an excitatory cue and an inhibitory cue. In contrast to the predictions made by models comprising a summed-error term (Rescorla and Wagner, 1972; Sutton and Barto, 1981), they found that learning was not distributed equally across the elements of the compound. When the compound was reinforced, the excitatory cue underwent greater change, however, when the compound was not reinforced the inhibitory cue underwent greater change. These data cannot be accounted for by a summed-error term (nor a differential degree of attention directed toward one of the cues). Rather, these data suggest that an individual-error term must be at least capable of contributing to associative change in some settings. As a consequence of such evidence, more recent developments in models of associative learning have taken into account the need for individual-error terms (Mackintosh, 1975; Pearce and Hall, 1980; Rescorla, 2000; LePelley and McLaren, 2004; Pearce and Mackintosh, 2010; Le Pelley et al., 2012). While there has been little investigation into the neural mechanism underlying individual-error terms, it would be of interest to identify whether midbrain dopamine signals may also reflect an individual-error term to contribute to associative change under these circumstances.

### How Might We Reconcile Evidence for Model-Based Learning in the VTA within the Current Framework?

Of course, a discussion of how VTA dopamine signaling impacts other structures to produce many forms of learning driven by error correction is a one-sided view. VTA dopamine neurons not only project out to a rich neural circuit, they receive dense reciprocal projections from these regions (Carr and Sesack, 2000; Vázquez-Borsetti et al., 2009, see **Figure 2**). Taking the broader circuitry into account, perhaps areas known to be involved in model-based reasoning inform VTA dopamine phasic signals of learning outcomes garnered from more flexible mental representations developed in the absence of direct experience. Thus, this information could be relayed in a top-down manner to VTA to modulate these phasic signals according to this word view (Daw et al., 2011; Takahashi et al., 2011, 2016; O’Doherty et al., 2017). However, it is also possible that VTA dopamine signals are causally involved in promoting the development of the associations which underlie the development of flexible mental maps which facilitate model-based inference. That is, these signals may provide more complex associative information about relationships between cues and outcome that facilitate model-based behaviors. While we have begun to scratch the surface of how dopamine signaling may influence model-based mechanisms, we need to start causally testing predictions of dopamine functioning beyond that envisioned by Sutton and Barto’s (1981) model-free reinforcement learning algorithm to truly understand all the weird and wonderful ways that phasic VTA dopamine supports associative learning.

## Author Contributions

HN and MS wrote major parts of the article. All other authors critically reviewed and edited the article. The review was written based on the expertise of the authors, who have sourced the article on PubMed and Google Scholar.

## Conflict of Interest Statement

The authors declare that the research was conducted in the absence of any commercial or financial relationships that could be construed as a potential conflict of interest.
